# The development of a multi-level intervention to improve data quality of the national quality indicators in Swiss long-term care facilities using an Intervention Mapping approach

**DOI:** 10.3389/frhs.2026.1796984

**Published:** 2026-05-11

**Authors:** Magdalena Osińska, Daniela Braun, Sonja Baumann, Brigitte Benkert, Nereide Curreri, Jianan Huang, Michael Simon, Bastiaan Van Grootven, Franziska Zúñiga

**Affiliations:** 1 Institute of Nursing Science, Department of Public Health, University of Basel, Basel, Switzerland; 2 Department of Business Economics, Health and Social Care, University of Applied Sciences and Arts of Southern Switzerland, Manno, Switzerland; 3 Department of Public Health, KU Leuven, Leuven, Belgium

**Keywords:** data quality, implementation science, intervention mapping, long-term care, nursing homes, quality indicators

## Abstract

**Introduction:**

Quality indicators (QIs) are used internationally to monitor performance and support continuous quality improvement. Their usefulness is dependent on the quality of the underlying data. Research indicates multiple barriers to reliable and accurate data collection in long-term care facilities (LTCFs). This paper describes the systematic development of an intervention to improve quality of QI data in LTCFs.

**Methods:**

The 6-step Intervention Mapping (IM) protocol guided the development process. 1) Needs assessment was done with an ethnographic study. 2) Intervention objectives were formulated followed by the selection of behavioral determinants. 3) Intervention design was based on evidence reviews, focus groups with LTCF staff and behavioral change methods. 4) Intervention materials and messages were developed in collaboration with stakeholders and external experts. We also developed 5) the implementation and 6) evaluation plan.

**Results:**

The needs assessment identified lack of awareness and knowledge, negative attitudes towards QI measurement, and environmental barriers. Having a person responsible for the topic, as well as monitoring and using QI data were seen as facilitators for data quality. The designed intervention uses a train-the-trainer approach. It consists of a training concept and support materials. The training aims to prepare champions to support their teams and lead data quality improvement activities: data quality monitoring, feedback, and internal staff trainings. The developed materials include a training handbook, lesson plans, presentations, factsheets, posters, and checklists. Other support measures included adapting QI measurement and providing an email contact for QI-related questions.

**Conclusion:**

The program theory and design rationale for this intervention will support the planned evaluation study, facilitate comparisons across similar interventions, and potentially inform the development of other interventions in LTCFs.

## 1 Introduction

Monitoring quality of care is crucial in face of increasing demands on health services due to demographic changes, increasingly complex healthcare needs, new advanced treatment options, and healthcare workforce shortages (1, 2). Quality indicators (QIs) are used internationally to monitor performance and support continuous quality improvement in healthcare institutions. QIs may not be absolute measures of quality, but they can still provide insights into it by describing the desired or undesirable structures, processes, and outcomes (3). Publicly reported QIs can provide governments, health insurance companies, care recipients, and the community with transparent information and assist decision-making (4, 5). As system-level measures of quality, they also inform policy and regulation. To be able to rely on the data provided by the QIs, healthcare professionals, care recipients, and governments need to be sure that QI results accurately reflect what they are supposed to measure. Moreover, if the data is used for benchmarking, QI results must be comparable across institutions (3, 6). Accordingly, data needs to be collected and recorded in a uniform way based on agreed-upon measurement instructions.

In residential long-term care facilities (LTCFs), QIs are often based on facility-reported clinical data (7), raising concerns about their quality. Studies in the U.S. uncovered that facilities use different strategies to improve their publicly-reported QI results, including modifying data collection and reporting processes, for example, by underreporting undesired outcomes (8, 9). Such practices lead to inaccurate or false information, undermine the trust in the results, and diminish the usefulness of the QI. LTCFs can also face various barriers to collecting, registering, and using QI data as intended, such as a lack of sufficient expertise, resources, or a perceived lack of influence on QI results (9). Such barriers should be identified and tackled to make the QI measurement effort meaningful and more likely to lead to actual improvements in the care provided to residents. However, little evidence is available about interventions improving data quality in LTCFs, and no explicit program theories exist (10).

In Switzerland, LTCFs are currently mandated to measure six clinical QIs in four topic areas related to polypharmacy, malnutrition, physical restraints, and pain. Their measurement is reported elsewhere (11). The QIs are calculated from routinely collected resident needs assessment data and reported yearly by the Federal Office of Public Health (FOPH). The reporting started with the cantonal-level data from 2019/2020, and the data from 2021 was published for the first time at the facility level (12). To promote quality of care in LTCFs, the government funded the National Implementation Programme: Strengthening quality of care in partnership with residential long-term care facilities for older people (NIP-Q-UPGRADE), aiming to investigate and improve data quality of the QIs, facilitate data-driven quality improvement, and introduce new QIs. Interventions developed and tested within NIP-Q-UPGRADE are intended for a national scale-up across approximately 1,500 LTCFs. The overall program follows the Exploration, Preparation, Implementation, Sustainment (EPIS) framework (13) and is further described elsewhere (14).

This paper describes the systematic development of a multicomponent intervention aiming to improve data quality of the current national QIs in Swiss LTCFs. Recognizing the complexity of the national LTCF setting and stakeholder interactions shaping QI data quality, we applied the Intervention Mapping (IM) approach (15) for intervention development. IM provides a structured planning framework for developing, implementing, and evaluating interventions, that integrates participatory methods and an ecological perspective to address problems and contextual factors at multiple levels. Transparent descriptions of the decision-making process and rationale at each step of designing, implementing, and evaluating the intervention can help identify the conditions that impacted its success or failure in the practice setting. It can also guide researchers considering replicating or adapting the intervention for other settings and facilitate comparison between interventions.

## 2 Methods

**Figure 1:**
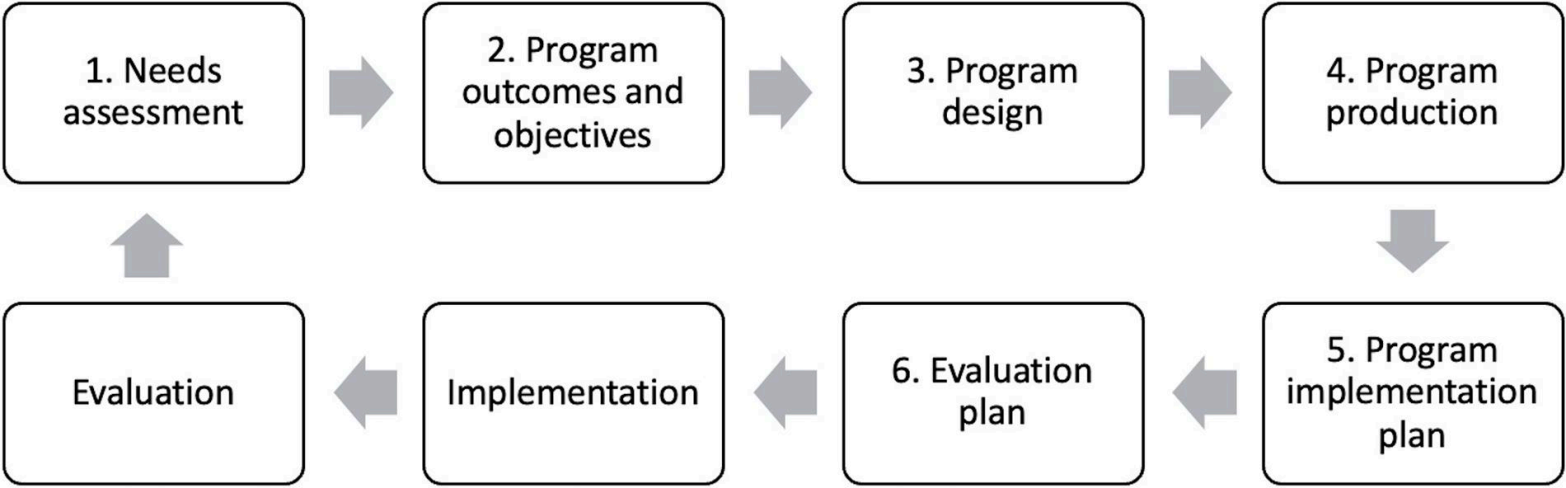
Intervention mapping steps.

IM is an intervention planning approach based on theory and evidence. It is characterized by an ecological approach to assessing and investigating problems and by engaging essential stakeholders in the design, planning, implementation, and evaluation of interventions aiming at behavior change. The IM process can be described as a step-by-step approach (cf. Figure 1) in which the outcomes of earlier steps inform later ones, although it is performed iteratively (15, 16).

IM consists of six steps (16). In step 1, a context and needs assessment is conducted. It includes identifying key stakeholders to be engaged, the behavioral and environmental causes of the problem, and the underlying determinants for the target population and the environmental agents. The selection of stakeholders was based on their expertise in long-term care, influence in the setting, responsibility in their organisations, interest in the project and availability. We also strived to include diverse perspectives e.g., of different country regions (17). The needs assessment allows to select specific target groups for the interventions. The deliverable of this step is a logic model of the problem. We performed a systematic literature review to identify factors related to data quality in LTCFs (18). Next, a rapid ethnographic study (19) was conducted in 2023 including observations and interviews with nursing and care staff members of 20 LTCFs across Switzerland, collaborating physicians, and pharmacists. In addition, we conducted focus groups with three providers of needs assessment software and seven providers of electronic health records (EHR), and asked for a software demonstration. This study captured how QI data is collected, registered, and managed by nursing professionals, and transferred to the Federal offices. It also identified factors that facilitate and hinder these processes in the context of Swiss LTCFs. A detailed report of this study is published separately (20).

In Step 2 of the IM, behavioral outcomes and performance objectives were formulated. They represent the (sub)behaviors that the target groups must perform to achieve the intervention goal. Key changeable determinants of behavior were selected based on the results of the needs assessment.

In step 3, the intervention design was conceptualized, including intervention components, change methods, and practical applications. To this end, existing theories and evidence on effective strategies to improve data quality in LTCFs were consulted. We reviewed evidence on interventions and strategies to improve data quality (10). In view of the planned scale-up, we also looked for evidence on strategies supporting scale-up process. To ensure the intervention adequately addresses the needs of the target groups, we conducted a qualitative study in a convenience sample of 28 LTCFs across Switzerland, including 3 main language regions. The study aimed to further assess what factors influence QI data collection in Swiss LTCFs, as well as support needs and preferences. A total of 107 participants including nurses, nurse experts, team leaders and higher management staff responsible for organizing or conducting QI assessments participated in 28 online focus groups. The discussions based on a semi-structured interview guide were moderated by a member of the research team, while another took notes. The discussions were video-recorded. Data was recorded directly in a dedicated summary template (21). Data collection took place between February and September 2024. Data were analyzed using inductive and deductive coding and thematic summaries.

In step 4, intervention structure and organization are further refined. To clarify the content of the intervention and materials, the research team established a working group: a nurse expert employed by one of the leading software providers, a quality leader, and a director of nursing, which allowed for in-depth discussions. Additionally, regional sounding boards with nurse experts and LTCF management persons in all language regions were consulted. The participants provided reflections on the results of conducted studies, their ideas, and feedback on the planned developments. For the development of the training concept, we collaborated with experts from an institution specializing in adult education. Materials were developed in collaboration with a company specializing in design and communication.

Step 5 concerns how implementation is planned to ensure that the intervention will be adopted, implemented, and sustained by the intended users. In this step we discuss also our approach to intervention scale-up, as a further implementation phase (16). Step 6 concerns planning the process and effect evaluation of the intervention. Considerations for these steps were made by the design of the NIP-Q-UPGRADE. These included selection of guiding implementation science frameworks, ensuring funding, defining dissemination strategies and identifying key partners in implementation, so that they could be involved not only in the implementation phase, but throughout the entire IM process (16).

## 3 Results

### 3.1 IM step 1 – needs assessment

The ethnographic study (18) revealed that knowledge about QIs among care staff is low. As the QIs are calculated from routinely collected data, care staff may not be aware of the QI data collection and how this data is used. Measurement instructions are often not followed, because they are either unknown or considered unclear. Some staff members have a negative attitude towards QI data collection, seeing it as an additional administrative burden that takes away their time with residents. They also perceive limited influence on QI results, particularly regarding polypharmacy, due to insufficient collaboration with physicians. Other identified barriers included low IT or language skills in some staff members, making the electronic documentation process challenging.

**Figure 2:**
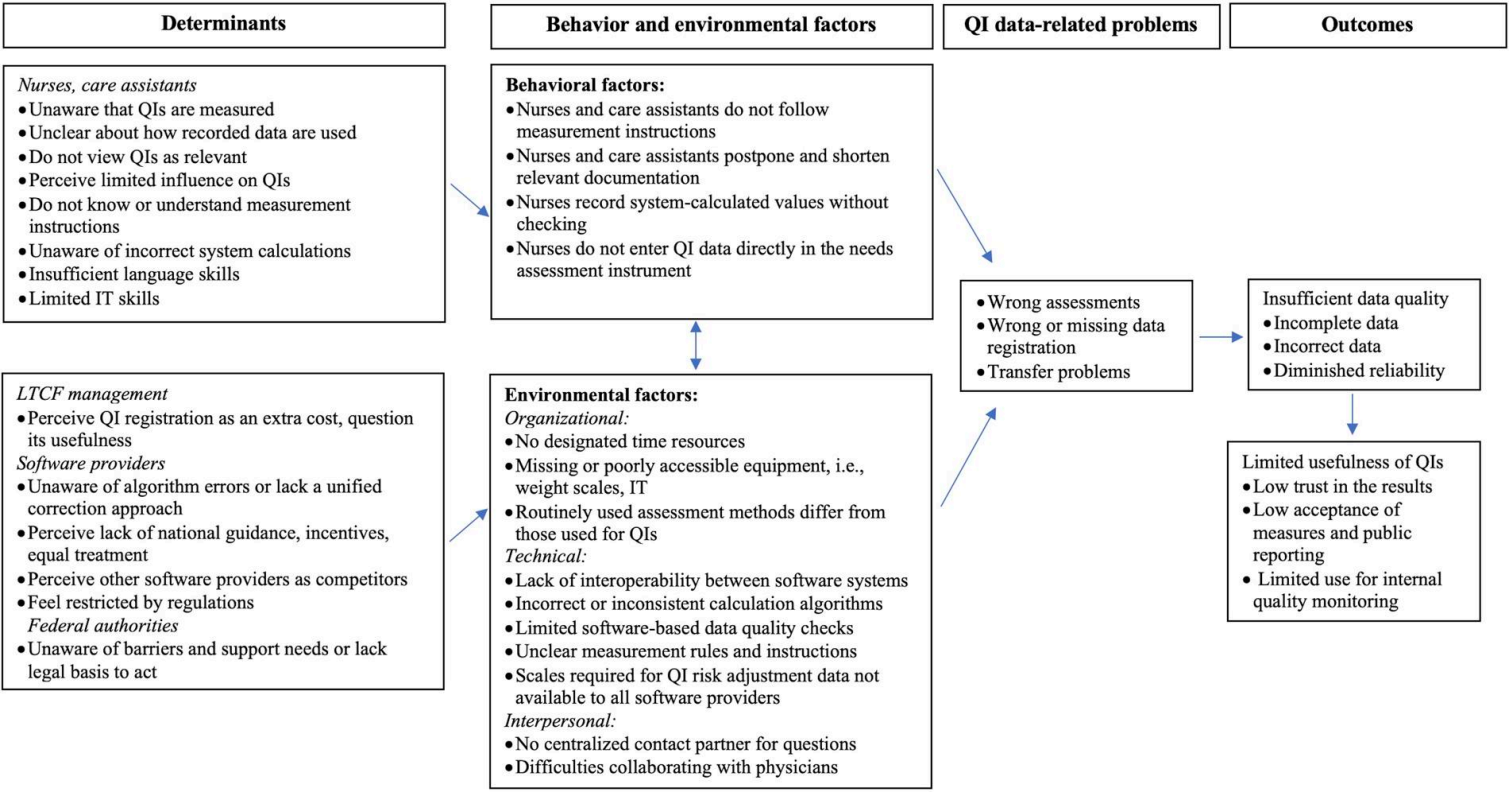
Logic model of the problem.

At environmental level, we learned that some LTCF managerial staff also do not consider QIs useful or essential for the practice, leading to a lack of willingness to allocate time resources and provide necessary equipment for data collection, also due to high workload and staff shortages in some facilities. Moreover, we identified a range of technical issues concerning the software used for QI data registration. Software providers use different algorithms for the calculation of weight loss and polypharmacy, which negatively affects the comparability of the data between facilities and causes over- or underreporting of the QIs. The lack of interoperability between resident documentation software and the needs assessment software leads to the necessity to transfer data manually, which increases the workload for data registration and the risk of errors. At the same time, few software providers implement features that enable automated data quality control, such as warnings when data is out of range. Figure 2 presents a logic model of the problem.

### 3.2 IM step 2 – intervention outcomes and objectives

We identified four target groups for the intervention at LTCF level: 1) registered nurses (RNs) and some licensed practical nurses (LPNs) who conduct assessments and record QI data in needs assessment or EHR software; 2) LPNs and nurse assistants who do not record QI data in the assessment instrument, but conduct and document some routine assessments that feed into the QI registration, e.g., weight; 3) LTCF management, and 4) staff with extended roles and/or specific responsibility for quality (e.g., quality managers, team leaders, nurse experts, needs assessment instrument experts). The targeted behavioral determinants for LTCF staff include the awareness and knowledge of the measurement, attitudes towards QIs, skills and self-efficacy in the assessments conducted for the residents, writing resident documentation pertaining to QIs, and registering QI items in resident needs assessment instruments. Key actors at the environmental level were the providers of needs assessment and EHR software, and the federal authorities. Though they were not the main intervention targets, their collaboration was deemed essential for tackling the environmental barriers to data quality. Table 1 describes behavioral and environmental outcomes and performance objectives defined for each group.

**Table 1:** Intended outcomes and performance objectives.

Outcomes	Performance objectives (expected behaviors)
RNs and LPNs with responsibility for QI assessment and recording assess and record QI data accurately.	Take up responsibility to assess and register QIs accurately Determine the correct assessment period Review resident documentation, e.g., medication list, weight or pain protocols, care notes Communicate with residents, relatives, team members, and physicians as needed to acquire information Document all information needed for QI assessments accurately Conduct assessment according to measurement instructions, i.e., using software, scales, observation Register assessment results accurately either directly in the needs assessment instrument or in EHR Transfer data correctly from EHR into needs assessment when needed
LPNs and nurse assistants without access to needs assessment instruments conduct and document assessments accurately.	Carry out weight and resident-reported pain assessment accurately Make observations related to signs of pain, depression, cognitive performance, and life expectancy ^a^ Communicate with residents, relatives, and team members as needed to exchange information Document assessments and relevant information accurately
LTCF management promotes accurate QI assessment and registration.	Decide to promote QI assessment and registration and communicate it to the staff Decide that QI assessment and registration processes are monitored and designate responsible staff Make arrangements to provide the time needed for QI assessment, registration, and data quality monitoring in work planning Provide resources for equipment needed for QI assessment and registration and training of staff on QIs Decide that processes and tools supporting accurate QI assessment and registration are implemented and designate responsible staff
LTCF staff responsible for quality development monitor and facilitate QI assessment and registration.	Decide to facilitate QI assessment and registration processes Monitor QI assessment and registration processes Include time needed for QI assessment and registration and data quality control in work planning (team leaders) Plan and provide regular training of staff on QIs Implement processes and tools supporting accurate QI assessment and registration into facility routines
Needs assessment instrument and EHR providers implement changes in the software to support correct QI assessment and registration.	Decide to further develop their software in a way that supports QI assessment and registration Implement changes in line with measurement rules Implement unified algorithms for calculations Design and implement data quality control and support features
The Federal Statistical Office and the Federal Office of Public Health support interventions for QI data quality improvement.	Recognize support needs of LTCFs and software providers Collaborate on adaptation of measurement and data recording rules and defining unified calculation algorithms Develop and disseminate information on correct QI data registration

EHR, Electronic Health Records; LTCF, Long Term Care Facility; QI, Quality Indicator; RN, registered nurse; LPN, licensed practical nurse.

^a^ Signs of depression, cognitive status are data elements used for risk adjustment; life expectancy under 6 months is used as an exclusion criterion in the calculation of QI malnutrition.

### 3.3 IM step 3 – intervention design

From focus groups with LTCF staff, we learned that having a dedicated person or group of people with a defined responsibility for QIs was seen as helpful for ensuring data quality. Some LTCFs already had such responsibilities assigned to particular roles, such as nurse experts or individuals specifically trained in the use of needs assessment software. They are tasked, for example, with monitoring documentation quality, answering questions on QIs, and coaching staff on assessments and data recording. Providing training opportunities was recognized as another facilitator, though QI training was previously conducted in only around 10% of participating LTCFs. Participants often expressed the wish for training to be delivered internally by a person with practical experience who knows how their LTCF is organized. Further, they pointed out that already existing knowledge gets easily lost due to staff turnover. Regarding support materials, we learned that participants often work with process descriptions, such as standards, protocols, and checklists in their practice. Key wishes were that the materials should be easily accessible, use visuals, and simple language. Some participants wished for interactive external assistance with QI questions via phone or e-mail. Other factors supporting data quality included data quality monitoring and providing feedback to the staff. Some LTCFs evaluate and exchange on QI results, which fosters understanding of the importance of reliable data. The findings of this study were in line with the literature on effective interventions and factors influencing documentation quality in LTCFs (10). In addition, systematic reviews indicate that strategies such as audit and feedback (22, 23), use of champions (24), and train-the-trainer models (25) can be effective in improving behavioral determinants and professional practice of healthcare staff. These strategies, along with setting up collaborations with stakeholders, are also often used in effective scaled-up or large-scale complex interventions in LTCFs (26).

Intervention components included the introduction of the role of a champion in the LTCF, who would lead activities aiming at data quality improvement: regular data quality monitoring and feedback on data collection and registration, internal training of care staff collecting QIs, and providing guidance, e.g., acting as a reference person for questions on QIs. Using a train-the-trainer approach, the research team developed a training concept for the champions and a set of support materials. Another support form was an email contact for champions and LTCF leadership. Theory and evidence-based change methods were consulted and integrated into the intervention components. Methods targeting the individual level were for instance facilitation, guided practice, and modeling (15), and are further described in step 4. By introducing the champion's role, we applied organizational change methods. Adding a new role and tasks can be seen as a structural redesign (15, 27). Part of the role is creating meaning of QIs and data quality (sense-making) in the LTCF (15, 28). Champions also provide an organizational diagnosis and feedback on data quality (15, 27).

The expected mechanism of change is that the champions are chosen from among nurses with existing responsibility for quality or for QI assessment and registration. They attend an external training, which helps develop the champion's role and implement data quality improvement tasks. The train-the-trainer approach promotes peer-to-peer learning and enables contextual adaptation, as champions know the local workflows and can tailor examples and explanations to their LTCF practice. Being part of the care team, they are likely to be trusted and approachable by the colleagues. Finally, train-the-trainer approach is potentially cost-saving and can help to build capacity to sustain the intervention beyond the initial implementation phase at individual organization and at a national level (25, 26, 29, 30). Support materials facilitate the learning and implementation process. Carrying out data quality monitoring allows for the identification of problems and training needs. Feedback to management makes the team's current level of knowledge transparent, and internal training sessions can be planned. Through feedback to the team, the champion can raise awareness of data quality issues and reinforce good practice. The champions are part of the team and act as role models and facilitators in daily practice. They encourage colleagues to reflect and ask questions. With individual feedback and internal training, staff members' knowledge, motivation, self-efficacy, and skills can be increased. These behavioral determinants are factors influencing implementation efforts and quality improvement at individual level (31, 32). The activities are expected to be repeated, engaging all relevant staff groups. This is intended to prevent knowledge loss and create a sense of importance, contributing to building a culture of quality (33) and leading to data quality improvement.

**Figure 3:**
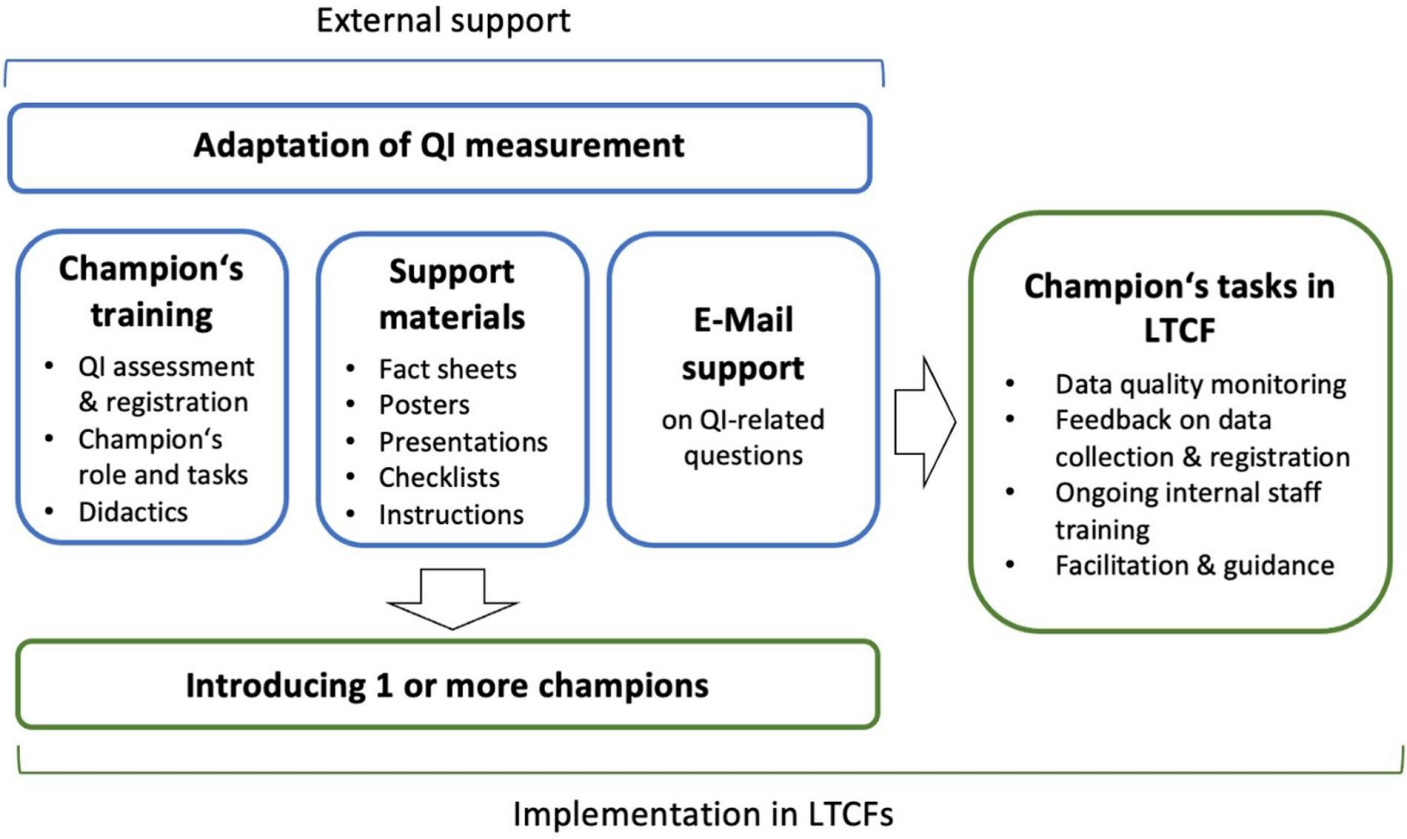
Intervention design.

For eliminating environmental barriers to data quality several meetings with resident needs assessment and EHR software providers, clinical experts, the Federal Statistical Office (FSO) and the FOPH members were conducted to foster interoperability, adapt QI measurement rules (e.g., for polypharmacy and pain observation), and develop uniform calculation algorithms (e.g., for weight loss and number of active ingredients administered to residents). These adaptations aimed to improve clarity, reduce the complexity of data collection, and the workload for the LTCF staff. These changes were achieved through participatory problem solving. Figure 3 provides an overview of the intervention design.

### 3.4 IM step 4 – intervention development

#### 3.4.1 Champions training concept

The initial training concept included 14 h of training, divided in 3 days, delivered in mixed format- onsite (8 h) and online (4 h and 2 h). The mixed format was intended to foster networking by meeting peers taking up the role in person, while also saving resources with online sessions. The intended delivery spreads over several months, to allow for implementation activities, such as clarifying the role, data quality monitoring and internal staff training between training days. The training was structured accordingly to deliver knowledge on the purpose and rules QI measurement, the delivery of training and feedback to the team. Practical tasks and support materials were introduced to develop skills and self-efficacy. Table 2 summarizes the main methods, practical applications, and materials used in champions' training.

**Table 2:** Methods, practical applications and materials used in champions’ training.

Sample performance objectives (PO) for the champions	Determinants	Sample methods and applications	Materials
PO1. Explain why and how QIs are measured and which factors influence data quality in their LTCF.	Awareness, Knowledge	Consciousness raising through communicating evidence. Modelling by trainers. Discussion with trainer and peers. Elaboration on the QI data. collection in own facility Facilitation through materials and e-mail support.	Preparatory task description, Training presentations, Factsheets
PO2. Decide to take up the champion's role.	Attitude, Knowledge, Skills and self-efficacy, Outcome expectations, Perceived norms	Arguments for the benefit of QI data quality Mobilizing social networks through onsite meeting and exchange with peers Facilitation through training and materials	Role description
PO3. Analyze inferences, barriers and facilitators to the role with the management.	As for PO2	Guided practice – practical task to discuss interfaces with the superior, sharing experiences in the next training session Discussion with trainer and peers Facilitation through materials	Practical task description Checklist to clarify interfaces
PO4. Explain the role to the stakeholders in their LTCF.	As for PO2	Guided practice Facilitation through materials	Practical task description
PO5. Conduct data quality monitoring.	As for PO2	Guided practice Facilitation through materials	Practical task description Checklist for data quality monitoring
PO6. Provide feedback to the management and team.	As for PO2	Knowledge provision by trainer Guided practice Facilitation through materials	Practical task description
PO7. Conduct internal trainings on QIs.	As for PO2	Knowledge provision by trainer Guided practice Facilitation through materials	Exemplary lesson plans presentations posters factsheets instructions on the use of materials
PO8. Plan further activities for data quality assurance.	As for PO2	Guided practice Discussion Goal setting	Practical task description

#### 3.4.2 Educational and support materials

The materials had different target groups. Materials for the deliverers of the champion's training included a handbook explaining the training concept, i.e., aims, target groups, and conditions, e.g., intended profile of the training deliverers. We developed also session outlines, PowerPoint presentations for each training session, and practical tasks descriptions to be handed out during the sessions.

Support materials for LTCFs aimed to facilitate the implementation of the new role and corresponding tasks. These were a checklist for clarifying the role and interfaces within the LTCF, champion's role description and candidate profile, a checklist for data quality monitoring, and instructions on the possible applications of educational materials. Educational materials included four factsheets, including updated measurement instructions for each QI, seven focused educational posters that contain information that can be read and absorbed in around one minute (34), PowerPoint presentations to facilitate internal training. The poster topics are: overview of the QIs, QI data cycle, as well as the aim and measurement of each of the six QIs. They can be printed and hung in places frequented by the staff. Five presentations cover an introduction to the topic of QIs and data quality, and each QI topic: polypharmacy, malnutrition, pain, and physical restraints. They are designed for 30–60 min. training sessions. Shorter educational inputs can be delivered based on the posters. The content is intended for care staff. It allows tailoring the internal training to the individual resources and needs of each LTCF. Factsheets contain a detailed explanation of the measurement and calculation of QIs, as well as how the QI topics can be approached in practice. The different materials together provide an opportunity for repeated exposure to the topics.

### 3.5 IM step 5 – implementation strategies

The Interactive Systems Framework (ISF) (35) indicates that interventions and implementation strategies are needed at different levels. The research team who summarized evidence and developed the interventions, can be considered a so-called synthesis and translation system. The LTCF staff (champions and management) are the delivery system, as they organize and implement interventions to ensure data quality. Between them, there is a support system, which should provide assistance, such as training and materials. In our case, the largest company providing needs assessment software in Switzerland became a partner in the initial champion's training delivery. The company has experience in organizing training for LTCFs on needs assessment and QI data collection. It became part of the support system by conducting the training and explaining the materials to the LTCFs. The strategies of the research team to support the training organization were participation in the intervention development process and delivery, facilitation through access to the dedicated materials for the champion's training, as well as to previously gathered evidence on existing needs, to raise awareness and knowledge. Further potential training organizations can be reached through project dissemination strategies. The LTCFs associations leading the NIP-Q-UPGRADE are an essential part of the support system. They promote the training and material through their channels and host a website where the developed materials are freely accessible for LTCFs (36).

Different dissemination strategies were planned. The LTCFs associations use outreach visits, conferences, webinars, newsletters and press communication (37) to disseminate the NIP-Q-UPGRADE and the developed resources among LTCFs and other stakeholders. The research team informs about the project via LinkedIn posts, at conferences, webinars, and via newsletters. The regional sounding boards are regularly updated about the developments. The key determinants targeted by the dissemination strategies are awareness and knowledge, attitudes, outcome expectations, and perceived norms. The methods used focus on information delivery and persuasive communication, through providing evidence from the conducted studies, and modelling through testimonials from trainers and champions who successfully implemented the interventions e.g., in a webinar reporting on the results of a pilot study.

Adoption, implementation and maintenance at LTCF level requires acceptance and active support of LTCF management (38, 39). Obtaining commitment of the management starts with selecting champions and sending them to the training. Throughout the training the champions are encouraged to seek support and engage their superiors in implementation activities, such as clarifying the role and interfaces, communication to the team, as well as planning and participation in internal trainings.

Strategies chosen as part of the intervention and implementation also foster sustainment. Involving key stakeholders in the planning and implementation process, building on existing systems and structures, engaging LTCF leadership to enhance their support, allowing for possibility to tailor implementation to individual needs of LTCFs and an ongoing access to the training for capacity building have been identified as sustainment strategies (40–42). Funding is a prominent factor influencing sustainability (40). Further attention is needed to ensure LTCFs have continuous access to champion's trainings at an affordable cost. Champions are key drivers of implementation and sustainment. Possibilities to foster sustaining networks between champions beyond the training to maintain knowledge exchange and positive attitude will be further explored. Our contextual analysis showed that ensuring high quality of care is a priority for LTCFs. (4) Continuously emphasizing the importance of ensuring data quality to be able to work with reliable data towards care quality improvement and better quality of life for the residents could maintain the alignment of the LTCFs' ultimate goal with the intervention and staff buy-in (40). This message is incorporated in project dissemination strategies, but also in staff trainings and on the posters. An overview of implementation strategies is provided in Table 3.

**Table 3:** Overview of implementation strategies.

Target group	Sample performance objectives	Determinants	Sample methods and applications
*Adoption*			
Software providers	Decide to implement adaptations in their software	Awareness Knowledge Outcome expectation	Participation in needs assessment study and consultation meetings on the adaptations of measurement Consciousness raising through dissemination strategies
Training organizations	Decide to organize trainings on QIs and data quality	Awareness Knowledge Outcome expectation	Consciousness raising through dissemination strategies Facilitation through materials on the website Modelling through testimonials
LTCF management	Identify/acknowledge need for intervention Select champions Send champions to training	Awareness Knowledge Outcome expectation Perceived norms	Consciousness raising and tailored messages through dissemination strategies Modelling through testimonials Facilitation through materials on the website
LTCF staff responsible for quality development	Identify need for intervention Bring it to attention of management	As for LTCF management	As for LTCF management
*Implementation*			
Software providers	Adapt interface and algorithms in line with updated measurement rules	Knowledge Outcome expectation	Facilitation through documents on adaptations
Training organizations	Organize trainings on QIs and data quality	Knowledge Outcome expectation	Facilitation through support materials on the website
Champions	Implement tasks for data quality improvement in LTCF	Knowledge Attitude Skills Self-efficacy Social support	Facilitation through champion's training and support materials Tailoring through choice of materials, frequency and duration of internal trainings
LTCF management	Clarify tasks and interfaces with champion Plan time resources Support champion in organizing staff trainings	Knowledge Outcome expectation Perceived norms	Facilitation, feedback, knowledge provision by the champion Tailoring through support materials
LTCF associations	Maintain website Scale intervention Monitor implementation	Knowledge Outcome expectation	Knowledge provision through reports with evidence and recommendations Facilitation through scale-up concept

Following evaluation and refinement the intervention is intended for a nationwide implementation. With this aim in mind, the design follows a phased scale-up process based on the model of Barker and colleagues (43). In the set-up phase, we designed the interventions and implementation strategies. The scalable unit development phase involves initial testing of the intervention as a scalable unit on a small scale to determine whether the developed resources work within the system and the local networks in three language regions (pilot study). In the test of scale-up phase, the scalable unit will be tested even more broadly in an effectiveness study. Going to full scale will be the final phase during which the knowledge, experience, skills, and infrastructure are in place to implement the intervention at full scale. Routine monitoring of intervention delivery is another strategy fostering sustainment (42). This could be done by proxy measures, such as monitoring the use of the website with intervention materials, monitoring requests for access to materials for training deliverers, or more directly by periodic surveys of LTCFs and training organizations. The research team will explore possible applications with stakeholders and give recommendations to the LTCF associations which will coordinate the scale-up process.

### 3.6 IM step 6 – evaluation plan

A pilot study assessing implementation outcomes, including acceptability, feasibility, fidelity, and costs, as well as perceptions on sustainability and scale-up was performed in 22 LTCFs across Switzerland. The study was conducted between January and June 2025. Data collection included surveys and focus groups with champions, LTCF management and nursing teams, interviews with the deliverers of the champion's trainings as well as activity logs. The study was registered in a clinical trials register (NCT06848725). The results and implications will be reported separately. An effectiveness study is planned for 2026 to assess the impact of the intervention on interrater reliability of the QI data (NCT06160024). Both evaluation studies will inform scalability of the intervention (44).

## 4 Discussion

We used IM and implementation science frameworks to develop an intervention to improve data collection of QIs in LTCFs. The intervention encompassed adaptation of QI measurement rules, a training concept and educational materials to facilitate introducing champions who would support the teams and lead data quality improvement activities: data quality monitoring, feedback and internal trainings.

The developed intervention uses a set of strategies and methods at different levels: LTCF organizations, individual LTCF staff members, software providers and federal authorities. This ecological perspective allowed us to address the problem of data quality holistically. Former interventions aiming to improve data quality in LTCFs often included only one component, such as a reminder strategy (assessment and documentation tool, checklist), educational meetings or materials, or a new electronic documentation system. Moreover, few studies report on the development or implementation process (10) limiting understanding of the context, change mechanisms and comparability.

IM was useful as a systematic and structured approach, that covers key aspects of implementation science and focuses on behavior change using evidence-based methods. However, as noted by other authors (45, 46), it is also complex and time intensive, especially in interventions with several target and implementer groups. The method uses a specific language, and requires extensive documentation, making it difficult to communicate, conduct and verify the steps with stakeholders or team members less familiar with the method. As many existing change methods can work on several behavioral determinants, and applications can include several methods the exact mapping process may be overwhelming. In our project, IM was largely followed as intended, with some adaptations and iterations to address contextual and practical considerations. In the IM protocol, theory-based methods are first mapped to change objectives, and then applications are chosen to deliver them (15). In our project, this sequence was inverted, because intervention components emerged clearly from the literature- and context-based evidence. As a result, the overall content was defined beforehand and subsequently checked against the theoretical methods and their parameters of use. As the champions were targets of the intervention and implementers at the same time, we had to formulate a separate set of performance objectives for this group, beyond those initially defined in step 2. Moreover, many considerations and decisions related to steps 5 and 6 – the implementation and evaluation plans – were made before the start of the formal intervention development process, as part of the overall National Implementation Programme. Finally, the IM protocol recommends establishing at least one planning group of key stakeholders that collaborates throughout the entire development process (15). Given the scope of our project, the diversity of stakeholders across three language regions, and time constraints, we opted instead to build on existing collaborations and to form smaller, task-focused groups.

The strengths of our approach were use of IM in combination with implementation science frameworks, including not only the development and implementation considerations, but also a scale-up plan. The development process involved an extensive formative work for needs assessment, identification of determinants and intervention components. We engaged a range of stakeholders and experts fostering understanding of the needs and collaboration at different levels. The developed intervention and implementation strategies focused not only on LTCF staff collecting data, but also on environmental agents representing organizations and the broader system. This approach allowed for designing tailored support methods and addressing key environmental barriers. However, our approach was resource and time intensive, and might not be feasible in other contexts. Working with several task-oriented groups might have limited the exchange of perspectives across stakeholder groups and decisions on the intervention content. Our research team had former expertise in implementation science, but not in the IM method. Though most team members attended a training on the method and we consulted external experts for the initial 3 steps of intervention design, we might not have used IM to its full potential. While following structured processes, and clear documentation of development steps are important for transparency and reproducibility, too much focus on adhering to methodological requirements may hinder co-design and limit the effective and efficient use of existing team expertise. Ideally, development teams should be led by researchers who combine contextual knowledge with expertise in co-design, IM process, and implementation science.

Nonetheless, this article provides a comprehensive description of the development process of an intervention for QI data quality improvement, to enhance transparency in view of a planned evaluation, as well as to foster possible adaptations and development of other behavior-change interventions.

## Data Availability

The datasets presented in this article are not readily available because the qualitative datasets (focus group and interview notes, and related materials) generated and analyzed for this study contain potentially identifiable and highly context-specific information about individuals and organizations. In line with the approved study protocol, participant consent, and applicable data protection regulations, these data cannot be shared outside the research team. Therefore, the datasets are not publicly available and cannot be provided on request. Requests to access the datasets should be directed to Franziska.zuniga@unibas.ch.
